# Attitudes of Polish physicians, nurses and pharmacists towards the ethical and legal aspects of the conscience clause

**DOI:** 10.1186/s12910-022-00846-0

**Published:** 2022-11-03

**Authors:** Justyna Czekajewska, Dariusz Walkowiak, Jan Domaradzki

**Affiliations:** 1 Department of Social Sciences and Humanities, Poznan University of Medical Sciences, Rokietnicka 7, St., 60-806 Poznan, Poland; 2 Department of Organization and Management in Health Care, Poznan University of Medical Sciences, Poznan, Poland

**Keywords:** Conscience clause, Conscientious objection, Ethical and legal aspects, Healthcare professionals, Nurses, Pharmacists, Physicians, Poland

## Abstract

**Background:**

While healthcare professionals’ right to invoke the conscience clause has been recognised as a fundamental human right, it continues to provoke a heated debate in Polish society. Although public discourse is filled with ethical and legal considerations on the conscience clause, much less is known about the attitudes of healthcare professionals regarding that matter. The aim of this study was therefore to describe the attitudes of Polish physicians, nurses and pharmacists towards the ethical and legal aspects of the conscience clause.

**Methods:**

We analysed a group of three hundred healthcare professionals: physicians, nurses and pharmacists in Poznan, Poland, using a standard questionnaire comprising of 29 questions about various ethical and legal aspects of the conscience clause and participants’ personal experiences with the conscience clause. The study was conducted between January and March 2020.

**Results:**

This research shows that although most Polish healthcare workers support the right to invoke the conscience clause they differ significantly in their opinions on to whom and to what medical procedures the conscience clause should apply to. It also demonstrated that while the conscience clause is rarely invoked in Poland, most healthcare professionals declare that the current legal regulations in that sphere are unclear and inaccurate.

**Conclusions:**

While there is an urgent need to raise the awareness regarding the conscience clause among medical students and healthcare professionals and educate them about such issues, it is even more important to improve the legal system in regard to the CC so that it protects both HCPs’ right to the CC and safeguards patients’ rights to medical services.

## Background

According to Polish legal regulations, healthcare professions (HCPs) are defined as independent professionals [1, 2]. Simultaneously, while professional independence reflects HCPs’ individual autonomy, autonomy is accorded by society and is based on trust [3]. For many years, therefore, the conscience clause (CC) in healthcare has provoked a heated debate in Polish society, as the majority of Poles believe that prenatal tests (73%), contraceptives (55%) and legal abortion (52%) should be exempt [4]. The number of publications on this topic has consequently increased significantly [5–13].

It is important, however, to distinguish between conscientious objection (CO) from the CC. The former is a moral norm which involves a persons’ refusal to comply with a particular order or rule because doing so entails betraying one’s moral reasons. Because conscience is equated with “moral integrity” or personal integrity, a person’s moral beliefs therefore reflect the moral core of their character, and thus preserving moral integrity proves the awareness of not violating moral obligations and is an important value in establishing the status of a moral person [14]. Thus, as a moral norm CO is the awareness of the existence of a judicial instance in man, that makes one responsible for the decisions she or he makes. It is derived from the freedom of conscience, and therefore the undertaken moral reflection should be free, as only in this way it determines the moral judgment of an individual. Moreover, the role of CO is to protect moral integrity, the right to freedom of thought, conscience, religion [13, 14], and the beliefs of every human being guaranteed by international documents on human rights, including the Universal Declaration of Human Rights [15], the International Covenant on Civil and Political Rights [16], and the European Convention on Human Rights [17]. The freedom of conscience has been also recognised and guaranteed by the constitutions of many European countries, including Germany [18], the United Kingdom [19], Portugal [20], and Poland [21].

On the other hand, the CC is a legal norm or clause attached to laws in various countries, including Poland, that exempts some individuals, including HCPs, physicians, nurses, or pharmacists, from providing certain services for moral or religious reasons [10, 22–26]. Nevertheless, the provision on the CC appears in the legislative provisions of many countries in order to prevent all forms of arbitrariness. For example, in Poland, HCPs who, for moral of religious reasons, object the provision of certain medical services, must first meet the applicable conditions set out in medical law, e.g. they cannot resign from performing professional activities in emergency situations which may pose a threat to the patient’s health or life; rather, the objector has to preemptively inform his or her supervisor and patient about the termination of any professional activity and is obliged to give legitimate reasons for one’s objection that should be noted in the medical documentation [27, 28].

Although an HCPs’ right to CO has also been recognised by the Council of Europe [29, 30], some authors argue that it is not absolute and should be monitored [24]. Since the CC offers HCPs the opportunity to refuse to perform certain medical procedures, it contrasts with patients’ right to healthcare services guaranteed by law. The opponents of CC therefore argue that, because it reduces patients’ access to appropriate medical treatment or services, it violates their right to medical services and may cause them harm [31, 32]. Since the imperative to protect patients’ health and life is safeguarded by most constitutions, it is often argued that HCPs’ right to the CC may cause harm in several ways, including delayed or restricted access to medical services, lack of important health information, additional healthcare expenditures and moral disapproval and stigmatisation of the patients’ choices. Therefore, Martin Benjamin and Julian Savulescu claim that all HCPs should be aware that if they are unable to perform certain medical procedures, they should resign from their jobs or switch to another area of healthcare [32, 33].

On the other hand, some suggest that, since the majority of cases in which HCPs invoke their right to the CC poses no risk to patients’ health or life, the objectors’ right to the freedom of conscience should be respected [6, 13]. For example, since most abortions are performed for social, financial, or partner-related reasons [34, 35], much fewer result from the mother’s or foetus’s health problems, and thus, it is argued that for the majority of cases invoking the CC, they are not associated with a threat to a woman's health or life [13, 36]. All in all, it seems that ethical and legal conflicts related to the CC result from the lack of reconciliation of the patients’ rights to healthcare services and HCPs’ rights to refuse to perform certain medical procedures that affront their moral or religious beliefs [11, 37, 38].

While physicians’ right to the CC was first recognised by the Polish Constitutional Tribunal in 1991 [6], it was included in the provisions of Polish medical law in 1996 and is currently regulated by art. 39 of the *Act on the Professions of Doctor and Dentist* [27] and art. 23 of the *Act on the Professions of Nurse and Midwife* [28]. It is also recognised by the *Code of Medical Ethics* (art. 7) [39] and *the Code of Professional Ethics of a Nurse and Midwife of the Republic of Poland* (art. 12) [40]. At the same time, although some argue that also pharmacists [41–43] and laboratory diagnosticians should be granted the right to the CO [44, 45], it applies only to physicians, nurses and midwives [7–13].

While all these documents stress HCPs’ right to refuse to participate in procedure or biomedical experiments that affront their moral or religious beliefs, in order to protect patients from the arbitrary refusal they impose on the objector the duty to give legitimate reasons for such a refusal.^1^ Crucially, as noted above, according to art. 30 of the *Act on the Professions of Doctors and Dentists* [27] and art. 19 of the *Act on the Professions of Nurse and Midwife* [28], the right to the CC may not be invoked in emergency situations that pose a threat to a patient’s life or may cause serious damage to their health. In such situations HCPs are obligated to provide the medical service required regardless of moral or religious beliefs. At the same time, on 7th October 2015 the Constitutional Tribunal declared unconstitutional previous regulations that imposed on the objector the duty to inform patients of the possible avenues they might pursue in order to receive the treatment refused by the objecting physician or nurse [46]. The amendment to the *Act on the Professions of Doctor and Dentist* was then promulgated on 7 October 2015 and art. 39 was changed accordingly, so that an HCP who currently invokes the CC need not indicate another person who will perform the medical procedure to which he or she objects. Thus, in its current form the document describes the CC as follows: *The doctor may refrain from performing health care services contrary to his conscience, subject to art. 30* [the doctor has a duty to provide medical assistance in any case, the delay in the granting of which could lead to danger of loss of life, serious injury or serious health disorder—J.C., D.W., J.D.]*, but he is required to record this fact in the medical records. The doctor carrying out his profession on the basis of an employment relationship or service has the obligation of prior written notification of his supervisor* [27]*.*

At the same time, while until 2020 abortion was legal in Poland on three conditions: when the woman’s health or life is in danger, when the pregnancy is a result of a criminal act (rape or incest) or on the grounds of severe or fatal foetal impairment or incurable life-threatening disease [49], in October 2020 the Polish Constitutional Tribunal declared that the third premise, or the so called “eugenic abortion” is unconstitutional, and since then it is no longer legal [36, 50, 51]. The Tribunal justified its decision by arguing that abortion for eugenic reasons discriminates against the unborn on the grounds of one’ health status and violates the dignity of disabled children and the right to life of every human being, which is guaranteed by Article 38 of the Polish Constitution [21]. Thus, abortion is now illegal with exceptions for the mother’s health or life, rape and incest.

In Polish medical practice HCPs most commonly object to performing legal abortions and prenatal screening tests which can help to detect any genetic or developmental defects in the foetus and can thus lead prospective mother or parents to choose to have an abortion. It is also used in the case of in-vitro fertilisation and when refusing to dispense contraception, including emergency postcoital contraception, the so called “morning-after pill”. As Poland is among those European countries where HCPs right to the CC is guaranteed by ethical codes and the medical law [24, 52], the aim of this study was to describe the attitudes of Polish physicians, nurses and pharmacists towards the ethical and legal aspects of the CC.

## Methods

The study was conducted between January and March 2020. Participating physicians, nurses and pharmacists were recruited during training sessions or specialisation courses organised by a variety of medical institutions in Poznan, Poland, including the Department of Gynaecological Oncology, Poznan University Hospital of Lord’s Transfiguration, the Poznan District Chamber of Nurses and Midwives and the Wielkopolska Regional Chamber of Pharmacy.

The survey was conducted by means of a standard questionnaire. It comprised topics derived from the literature review. It consisted of three main sections. The first collected HCPs demographic data regarding sex, age, profession, seniority, domicile and religious beliefs. The second section included questions regarding the legal aspects of the CC in Poland. The last section focused on participants’ personal experiences with the CC.

The study was performed in line with the principles of the Declaration of Helsinki. Ethical approval was obtained from the Wielkopolska Regional Chamber of Pharmacy in Poznan (Wlkp. OIA/2711/2018), from the Poznan District Chamber of Nurses and Midwives (OIPIP 15/3/2019) and the from the Department of Gynaecological Oncology, Poznan University Hospital of Lord’s Transfiguration (LBK/63/2018). Approval of the research governance was obtained from the Poznan University of Medical Sciences’ Bioethics Committee. Informed consent was obtained from all respondents enrolled in the study.

The data collected in the questionnaires were verified and checked for completeness, quality and consistency and exported into the statistical package STATISTICA v. 13.3 (TIBCO, Palo Alto, USA). The results are presented as descriptive statistics, means and standard deviations (SDs) and percentages of groups. Kruskal–Wallis ANOVA followed by post hoc tests were used to determine statistically significant differences between the three groups of HCPs. We set up the level of significance at *p* < 0.05. In the case of small group size (< 15), the differences between groups were not determined.

## Results

The group consisted of three hundred HCPs in equal proportions, one hundred from each of the professional groups: physicians, nurses and pharmacists (Table 1). While the majority of respondents were women (81%), among the nurses this ratio was 98%. The overrepresentation of women may, however, be explained by the fact that many medical professions in Poland are dominated by women [53]. As many as 271 of the respondents had higher education, but in the case of physicians, who must obtain the license to practice medicine, all respondents necessarily hold a university degree. Although 43.7% of the respondents declared themselves practicing believers, 33% declared themselves to be non-practicing believers. 38.3% of the respondents separated religion from public issues and declared that it played no significant role in their life.

**Table 1:** Socio-demographic characteristics of the respondents and their attitude to religion

Characteristics	1. Physicians n (%N)	2. Nurses n (%N)	3. Pharmacists n (%N)
Number of respondents	N = 100	N = 100	N = 100
Sex			
Woman	71 (71)	98 (98)	74 (74)
Man	29 (29)	2 (2)	26 (26)
Mean age	33.8	40.2	31.9
Minimum age	25	25	22
Maximum age	61	59	59
SD	6.6	10.2	8.5
Education			
University	100 (100)	85 (85)	84 (84)
High school	0	15 (15)	16 (16)
Religious practices			
Believing/practicing	44 (44)	63 (63)	24 (24)
Believing/not practicing	26 (26)	29 (29)	44 (44)
Non-believer/practicing	3 (3)	0	7 (7)
Non-believer/not practicing	27 (27)	8 (8)	25 (25)
What role does religion play in your life?			
Significant; it influences my life decisions and choices	10 (10)	21 (21)	12 (12)
Rather significant; I try to follow religious principles in my life	30 (30)	40 (40)	15 (15)
Insignificant; I separate religion from public issues	31 (31)	30 (30)	54 (54)
None; it is irrelevant to me	29 (29)	9 (9)	19 (19)

Most respondents declared that the CC should apply to abortion, contraceptives and assisted reproduction techniques (Table 2). In all cases physicians were most likely to support HCPs’ right to the CC, followed by nurses, while pharmacists were the least likely to do so. For example, while 94% of physicians and 95% of nurses believed that HCPs should have the right to refuse to perform an abortion, only 72% of pharmacists shared this opinion. On the other hand, respondents’ opinions on contraceptives varied: while 68% physicians supported this application of the CC, nurses and pharmacists did so less frequently (42% and 38% respectively).

**Table 2:** Respondents’ opinions on medical procedures that should be covered by the CC

	1. Physicians n (%N)	2. Nurses n (%N)	3. Pharmacists n (%N)	*p* for groups differences
Abortion	94 (94)	95 (95)	72 (72)	1 vs. 3 *p* < 0.001 (*) **2 vs. 3 *p* < 0.001**
Prenatal testing	47 (47)	22 (22)	20 (20)	**1 vs. 2 *p* < 0.001** **1 vs. 3 *p* < 0.001**
Contraception	68 (68)	42 (42)	38 (38)	**1 vs. 2 *p* < 0.001** **1 vs. 3 *p* < 0.001**
Assisted reproduction techniques	51 (51)	43 (43)	33 (33)	**1 vs. 3 *p* < 0.01**
Transplantology	39 (39)	27 (27)	14 (14)	**1 vs. 3 *p* < 0.001** **2 vs. 3 *p* < 0.05**
Palliative care	53 (53)	26 (26)	21 (21)	**1 vs. 2 *p* < 0.001** **1 vs. 3 *p* < 0.001**
None of the above	7 (7)	4 (4)	27 (27)	**1 vs. 3 *p* < 0.001** **2 vs. 3 *p* < 0.001**

Results shown exclusively for significant differences

*Statistically significant values are written in boldfaces

While physicians indicated palliative care and prenatal testing as legitimate causes for invoking the CC more often than other professional groups, their opinion on transplantology differed only insignificantly from that of nurses.^2^ At the same time, both these groups indicated transplantology as a legitimate cause for invoking the CC more often than did pharmacists.

Based on the responses regarding HCPs’ attachment to religion and its role in their life, we divided them into two groups: religious (respondents who declared that religion influences their life decisions and choices or those trying to follow religious principles in their lives) versus nonreligious (respondents who were not attached to religion or felt it was irrelevant to them). The largest percentage of people declaring themselves religious was found in the group of nurses, while physicians were the second most religious group. However, it should be noted that there was no statistically significant difference between the percentages of respondents classified as religious in the groups of physicians and pharmacists. While 21% of nurses declared that the CC should apply to all HCPs, only 4% of physicians and pharmacist granted this right to both physicians, nurses and pharmacists (Table 3). Indeed, physicians believed that the CC should apply primarily to their professional group. On the other hand, while nurses indicated to physicians (65%) and nurses (64%) most often, pharmacists pointed to physicians (38%). Few respondents across the groups indicated pharmacists (11% of physicians, 15% of nurses and 17% of pharmacists).

**Table 3:** Respondents’ opinion on whom the conscience clause should apply

	1. Physicians n (%N)	2. Nurses n (%N)	3. Pharmacists n (%N)	*p* for groups differences
Respondents				**1 vs. 2 *p* < 0.01** **2 vs. 3 *p* < 0.001**
Religious	40 (40)	61 (61)	27 (27)	
Nonreligious	60 (60)	39 (39)	73 (73)	
Conscience clause should apply to:				**1 vs. 2 *p* < 0.001** **1 vs. 3 *p* < 0.05** **2 vs. 3 *p* < 0.001**
All HCPs	4 (4)	21 (21)	4 (4)	
Some groups of HCPs	57 ((57)	65 (65)	38 (38)	
Nobody	39 (39)	14 (14)	58 (58)	
CC should apply to:				**1 vs. 2 *p* < 0.001** **1 vs. 3 *p* < 0.05** **2 vs. 3 *p* < 0.001**
Physicians	57 (57)	65 (65)	38 (38)	
Nurses	45 (45)	64 (64)	26 (26)	
Pharmacists	11 (11)	15 (15)	17 (17)	
Other medical professionals	0	2 (2)	1 (1)	

Statistically significant values are written in boldfaces

Less than 10% of the HCPs in the study have ever invoked the CC (Table 4). 20% of physicians, 13% of nurses and 6% of pharmacists, however, expressed a willingness to do so. The vast majority of those who considered invoking the CC were concerned that to do so may lead to their losing their jobs or that the decision could harm their relationships with other HCPs. At the same time very few respondents declared having ever been forced to perform a medical service which affronted their conscience.

**Table 4:** HCPs personal experiences with the conscience clause

	1. Physicians n (%N)	2. Nurses n (%N)	3. Pharmacists n (%N)	*p* for groups differences
Have you ever invoked the conscience clause?				ns
Yes	3 (3)	9 (9)	6 (6)	
No	97 (97)	91 (91)	94 (94)	
If so, did you have any concerns about it?				
Yes	2 (66.7)	6 (66.7)	3 (50)	
No	1 (33.3)	3 (33.3)	3 (50)	
Have you ever considered invoking the conscience clause?				**1 vs. 3 *p* < 0.01**
Yes	20 (20.6)	13 (14.3)	6 (6.4)	
No	77 (79.4)	78 (85.7)	88 (93.6)	
Were you concerned about this?				
Yes	17 (85)	11 (84.6)	4 (66.7)	
No	3 (15)	2 (15.4)	2 (33.3)	
If so, what were your concerns?				
Losing job	15 (78.9)	11 (64.7)	4 (57.1)	
Potential difficulties in achieving promotion	7 (36.8)	0 (0)	0 (0)	
Relationships with other employees	13 (68.4)	13 (76.5)	2 (28.6)	
Losing respect	11 (57.9)	2 (11.8)	4 (57.1)	
Other concerns	2 (10.5)	2 (11.8)	1 (14.3)	
Have you ever been in an emergency situation where it was necessary to perform a medical service that affronted your conscience?				ns
Yes	19 (19)	15 (15)	18 (18)	
No	81 (81)	85 (85)	82 (82)	
If so, in what situation?				
Participation in the termination of pregnancy	5 (26.3)	3 (20)	0 (0)	
Performing the termination of pregnancy	5 (26.3)	0 (0)	0 (0)	
Participation in a blood transfusion procedure	0 (0)	2 (13.3)	0 (0)	
Carrying out the blood transfusion procedure	0 (0)	2 (13.3)	0 (0)	
Participation in biomedical experiments	2 (10.5)	2 (13.3)	0 (0)	
Carrying out biomedical experiments	0 (0)	0 (0)	0 (0)	
Dispensing drugs	7 (36.8)	9 (60)	18 (100)	
Other	5 (26.3)	2 (13.3)	0 (0)	
Would you object to a pharmacist’s refusal to fill a prescription for contraceptives available in pharmacies?				**1 vs. 2 *p* < 0.01** **2 vs. 3 *p* < 0.01**
Yes	81 (81)	64 (64)	77 (77)	
No	16 (16)	23 (23)	22 (22)	
I have no opinion	3 (3)	13 (13)	1 (1)	
I would object to a refusal to fill the following contraceptives				**1 vs. 2 *p* < 0.01** **1 vs. 3 *p* < 0.01**
Hormonal contraceptives	76 (100)	51 (94.4)	47 (95.9)	
Mechanical methods of contraception, e.g. vaginal rings, condoms	76 (100)	49 (90.7)	39 (79.6)	
Chemical barrier methods, e.g. globules, foams, creams, spermicidal fluids	76 (100)	46 (85.2)	43 (87.7)	
Methods that prevent fertilisation, e.g. IUD	76 (100)	34 (63)	35 (71.4)	
Postcoital contraception (the “morning-after pill”)	63 (82.9)	24 (44.4)	31 (63.3)	

Statistically significant values are written in boldfaces

Nurses differed significantly in terms of statistics from both physicians and pharmacists in their opinions of a pharmacist’s refusal to fill a prescription for contraceptives available in pharmacies. Such a refusal was disdained by 81% of physicians, 77% of pharmacists but only 64% of nurses.

While most physicians (67%) and pharmacists (73%) believed that the law should oblige HCPs who refuse to perform a medical procedure due to their moral objections to refer the patient to another specialist, nurses were least likely to support such a regulation (39%) (Table 5). Moreover, while Polish law clearly prohibits an employer from inquiring about one’s philosophy of life, religious convictions, or beliefs [21, 54], physicians enrolled in this study also believed that a prospective employer should be entitled to ask candidates about their private beliefs regarding the CC during a job interview. They also declared that the current law fails to describe with sufficient precision the rules regarding HCPs invoking the CC. Finally, 88% of physicians, 85% of nurses, and 75% of pharmacists declared that topics regarding the CC should be discussed more in-depth during classes on medical ethics and philosophy.

**Table 5:** Respondents’ opinions on legal, formal and ethic aspects of conscience clause

	1. Physicians n (%N)	2. Nurses n (%N)	3. Pharmacists n (%N)	*p* for groups differences
Should the law oblige HCPs who refuse to perform a medical procedure due to ethical objections to refer the patient to another specialist?				**1 vs. 2 *p* < 0.001** **2 vs. 3 *p* < 0.001**
Yes	67 (67)	39 (39)	73 (73)	
No	27 (27)	37 (37)	18 (18)	
I have no opinion	6 (6)	24 (24)	9 (9)	
Do you think that a prospective employer should be entitled to ask candidates about their private beliefs about the CC during a job interview?				**1 vs. 2 *p* < 0.001** **1 vs. 3 *p* < 0.05** **2 vs. 3 *p* < 0.05**
Yes	54 (54)	26 (26)	36 (36)	
No	43 (43)	50 (50)	54 (54)	
I have no opinion	3 (3)	24 (24)	10 (10)	
Do you think that the current law precisely describes a set of rules established for HCPs who use the CC?				**1 vs. 2 *p* < 0.001** **1 vs. 3 *p* < 0.05**
Yes	6 (6)	14 (14)	8 (8)	
No	75 (75)	44 (44)	59 (59)	
I have no opinion	19 (19)	42 (42)	33 (33)	
If not, what remains not fully regulated?				ns
Determining the scope, i.e. determining which group of HCPs has the right to invoke the CC	53 (70.7)	27 (61.4)	42 (71.2)	
Establishing the conditions that must always be met if an HCP wants to invoke the CC	64 (85.3)	36 (81.8)	50 (84.7)	
Measures to ensure that the patient has practical access to a specific health service	59 (78.7)	25 (56.8)	46 (78)	
All of the above-mentioned circumstances	45 (60)	16 (36.4)	34 (57.6)	
Other	2 (2.7)	0 (0)	1 (1.7)	
Is it necessary to discuss the CC during classes in ethics and philosophy during medical studies?				ns
Yes	88 (88)	85 (85)	75 (75)	
No	5 (5)	4 (4)	9 (9)	
I have no opinion	7 (7)	11 (11)	16 (16)	

Statistically significant values are written in boldfaces

## Discussion

Over the last three decades the CC has become one of the most controversial medical topics both among HCPs and within Polish society [4], especially insofar as the European Court of Human Rights has ruled in several cases that HCPs’ invoking the CC has resulted in the denial of women’s right to reproductive care (i.e. Tysiąc v. Poland, R. R. v. Poland and P. and S. v. Poland^3^). It also ruled that Poland’s duty ensure that women’s access to reproductive services, including prenatal testing or legal abortion (i.e. when there is an assumption of foetal malformation, when there is a health risk to the mother or when the pregnancy is a result of rape) does not infringe HCPs’ right to invoke the CC [55–58].

The CC gained ground in May 2014, when almost 3,000 Polish physicians, including 59 professors of medicine and medical students, signed the *Declaration of faith of Catholic doctors and students of medicine, on the sexuality and fertility of human beings*,^4^ in which they stressed the primacy of “God’s law” and religious beliefs on the human body, life, and reproduction over the national legal regulations, and considered abortion together with other reproductive services as an affront to their conscience [59]. Even though the number was rather small, as at that time there were more than 230,000 practicing physicians (excluding dentists) and more than 7,000 medical students in Poland [60], still it provoked the heated debate on the CC and increased the level of anxiety in society, as the majority of Poles declared that HCPs should be forbidden to refuse to perform such medical procedures as prenatal tests, legal abortion, or prescribe contraceptives [4].

Although many Poles still define themselves as Catholics, over the years the number of people who define themselves as believers declined from 96% in 2005 to 91% in 2020. Simultaneously, the number of those who define themselves as nonbelievers has increased from 5 to 9% respectively [61, 62]. While most HCPs enrolled in this study declared themselves to be practicing Christians, still over one third of respondents separated religion from public issues and declared that it played an insignificant role in their life. Many respondents therefore seem to reject the official teachings of the Catholic Church on such bioethical issues as contraceptives, abortion, or assisted reproduction techniques, and are guided more by secular values. These changes are the result of social, economic, and political transformation that began in Poland in the 1990s and raised people’s awareness of the importance of personal freedom and maintaining one’s own views and personal distinctiveness [63, 64]. Focusing attention on freedom of choice was the reason for accepting respect for social and cultural differences and recognition of the equality of views [64, 65]. Thus, while some stress the impact of the Catholic moral theology on the formation of CO, it is not exclusively inherent to the religious sphere as it derives from freedom of conscience and is a product of liberal and libertarian philosophy.

This research shows that the majority of respondents see the need to perform professional duties in accordance with their conscience and support physicians’ and nurses’ rights to the CC. Similar results were obtained by Alouini and colleagues, who showed that 65% of gynaecologists from 16 countries were also against the liberalisation of abortion laws in their countries and declared that their personal beliefs on abortion were more restrictive than their national legal regulations [66]. In Italy 70% of HCPs, including practicing physicians, nurses and midwives, as well as medical students also support the right to the CC [22, 67]. Over 45% of medical students in the United Kingdom [68], 79.2% in Spain [69] and 89% of Slovak pharmacists and pharmacy students also support this right [26]. Finally, a previous study conducted among Polish pharmacists demonstrated that 65% of pharmacy students and 50% of practising pharmacists support the introduction of the CC for pharmacists to Polish medical law [25].

This study also suggests that the majority of Polish HCPs support a narrow scope of circumstances that offer the right to invoke the CC and declared that in accord with current Polish law the CC should apply to physicians and nurses and should exclude pharmacists. For example, a study conducted by Piecuch, Gryka and Kozłowska-Wojciechowska among Polish pharmacists showed that 73% of respondents believed that the right to the CC should exclude pharmacists [42]. Baranowska and colleagues demonstrated that pharmacists are more likely to refuse to grant the right to CC to their fellow pharmacists [41]. Finally, Czekajewska, Langer and Baum showed that, according to pharmacists, invoking the CC would hinder patients’ rightful access to various pharmaceutical services, undermine their trust in HCPs and generate moral conflicts with other employees and patients [12].

This study is also in line with findings from others that have showed that the most common reasons for using HCPs right to the CC is abortion and assisted reproductive techniques [26, 42, 67]. In contrast to previous Polish research, however, our respondents also indicated contraception as the second most common reason HCPs should be entitled to invoke the CC. Emergency postcoital contraception (the so-called “morning-after pill”) and intrauterine devices were therefore indicated as products that pharmacists should have the right to refuse to sell because of conscientious objections. This confirms the findings from Piecuch, Gryka and Kozłowska-Wojciechowska’s study in which they showed that according to Polish pharmacists medicinal products that should fall under the CC are primarily emergency contraception (65%), vaccines (46%), intrauterine devices (27%) and hormonal contraception (21%) [42, 70, 71]. Similar results were also obtained in other European countries, including the United Kingdom, Italy, Portugal and Norway, which have similar CC regulations [24, 43].

The right to invoke the CC is rarely exercised by HCPs. Durak and colleagues, however, also showed that, while most nurses in Lublin experienced moral conflict at work, 84% have never exercised their right to invoke the CC [72]. Similarly, according to Piecuch, Gryka and Kozłowska-Wojciechowska [42], while 92% of Polish pharmacists have never refused to fill a prescription because of their beliefs, only 15% would use this right if medical law allowed it. Similarly, only 32.5% of Slovakian pharmacists declared having invoked the CC at work [25].

Finally, this study suggests that, while HCPs want to perform their work in harmony with their conscience, in situations of moral conflict most of them prefer to surrender their right to the CC because they are afraid of possible conflicts at work [72, 73]. Respondents enrolled in this study therefore stressed that, while the use of the CC resolves no conflicts, it may provoke conflict in relation with fellow HCPs and patients. Most of these concerns relate to invoking the CC in situations that do not follow from the act and restrict patients’ access to legal medical service. According to Baranowska and colleagues 76% of pharmacy students and 43% of practicing pharmacists also declared that, if the law allows pharmacists to invoke the CC, it would limit patients’ freedom of choice [41]. This is supported by Montgomery [23], who argues that ensuring patients’ free access to comprehensive and non-discriminatory medical services is a clear priority for all HCPs. Dickens [37] also suggests that a patient’s right to health is paramount and may therefore not be subject to an HCPs’ personal beliefs [32].

HCPs feel responsible for the decisions they make, so they seek to perform their professional duties in accordance with the beliefs of their patients. Moral conflicts will continue to exist in the workplace, but this is no reason to ignore the problems that arise from conflicting views.

As our study shows, HCPs feel that there is a need for education in this area and believe that the issues of the CC should be discussed more in-depth during medical ethics, bioethics, medical philosophy, and medical law classes. Cooper et al. [74], Crespigny and Savulescu [75], and Lamb et al. [73, 76] also found that HCPs see the need to expand their knowledge of the ethical and legal issues related to conscientious objection.

All in all, this research shows that, while according to HCPs the opportunity to perform their professional activities in accordance with their conscience is an important individual right, they also declared that the CC should be legally restricted when it constitutes the imposition of HCPs’ personal moral convictions on the patient. They therefore believed that the CC may pose a risk to patients’ health or life and may damage HCPs’ relationships with co-workers and patients, and reduce the latter’s’ confidence and trust in HCPs.

### Study limitations

This research has a number of limitations. Firstly, the sample was rather small and this may have an impact on the generalisability and interpretation of the results. The research was, however, conducted during the COVID-19 pandemic, which hindered the recruitment process and reduced the number of respondents. Secondly, as it covered HCPs from only one Polish city, the study has a local dimension and the results cannot be extrapolated to include all HCPs either in Poznan or in Poland as a whole. Thirdly, as the results are mainly descriptive, it is impossible to determine the causality of certain factors. Future studies should therefore compare the findings from larger groups and from other parts of Poland. Finally, as this study is based on the quantitative method only, to better understand HCPs lived experiences with the CC, further in-depth studies using qualitative methods would be required. The unquestionable strength of this study is nevertheless fills the gap in the research on the attitudes of Polish HCPs towards the CC.

## Conclusions

While this study has demonstrated that HCPs in Poland support the use of the CC, it has also showed that physicians, nurses and pharmacists differ in their opinions regarding those to whom and the medical procedures to which the CC should apply. While physicians and nurses were more likely than pharmacists to support the CC, they were also keener to grant this right to other HCPs. On the other hand, all HCPs declared that the CC should apply to abortion, contraception, methods of assisted reproduction and palliative care.

Another important finding is that, while the CC is rarely invoked in Poland, most physicians, nurses and pharmacists who participated in the study believed that the current legal regulations regarding the CC are unclear and vague. As they suggested that the law should clearly determine those who should have the right to refuse treatment and the circumstances under which they may do so, they also emphasised that it safeguard patients’ right to healthcare services.

Finally, our results indicate that, because most HCPs felt confused to some extent about the ethical and legal issues related to the CC, there is an urgent need to raise awareness regarding the CC among medical students and healthcare professionals and educate them about such issues. Respondents declared that one way to improve this is to change the curricula at medical schools and that the CC should be discussed during ethics and philosophy classes. Postgraduate specialisation courses on medical ethics, moral psychology and medical law should also be organised in order to help HCPs develop the practical skills required for solving ethical problems they encounter during their professional practice. Because most HCPs lack time, e-learning modules containing lecture videos, case studies, webinars and PowerPoint presentations should be organised. Finally, the Polish Chamber of Physicians and Dentists, the Wielkopolska Regional Chamber of Pharmacy and the Poznan District Chamber of Nurses and Midwives should establish “ethical support units” to help HCPs make difficult choices in situations of moral conflict.

However, because most HCPs enrolled in this study believed that the current legal regulations on the CC in Poland are not clear enough and therefore can become a source of conflict between HCPs and patients, it is even more important to improve the legal system in regard to the CC so that it protects both HCPs’ right to the CC and safeguards patients’ rights to medical services. For example, according to certain legal acts, including The United Nations Human Rights Committee’s on the fifth periodic report of Poland (CCPR/C/POL/2004/5) [77] and The Committee on the Elimination of Discrimination against Women, which considered Poland’s sixth periodic report (CEDAW/C/POL/6) [78], Polish physicians employed in public health units, relying on the CC, neither agree to perform an abortion even when women meet the legal criteria nor do they provide them with information on where to go to receive the medical benefit due to them. Thus, as suggested by Różyńska [79], since the CC regulations in Poland are still the subject of doctrinal discussions, interpretational doubts concerning the following issues should be solved: the normative nature of an HCP’s right to refuse to perform a medical service, determining medical services that should be covered by the CC, determining situations in which an HCP is not entitled to use the CC, and defining obligations both towards the patient and the employing entity, which should rest on withdrawing the HCP from the performance of a health service.

## Data Availability

The datasets generated during and/or analysed during the current study are available from the corresponding author on reasonable request.

## Abbreviations

CC: Conscience clause
CO: Conscientious objection
HCP: Healthcare professionals

## Footnotes

1 According to Polish regulations there are two main reasons why HCPs can appeal to the CC: (1) HCPs can refuse to provide services that are not of a therapeutic character or are only partially therapeutic and require sacrificing one legally protected good by another (i.e. abortion, transplantation from a living donor); (2) HCPs can refuse to provide services that do not require sacrificing one legally protected good by another, but are contrary to one’s conscience or beliefs (i.e. blood transfusion in case of Jehovah’s Witnesses, performing IVF or IUD placement by a Catholic physician) [47]. According to Szewczyk, the main rationale for this being that while a physician can rely on her or his personal conscience, there is no such thing as “institutional conscience” that could be applied to an entire medicine or healthcare system. Thus, a physician cannot argue that a particular medical procedure or service she or he refuses to perform is inconsistent with medical ethics [48].

2 The main ethical issue in palliative care relates to consistent therapy. Since every patient has the right to a natural, dignified, and conscious death, HCPs can refuse to continue futile medical care when a family insists on supporting a patient’s vital functions even though it only extends his or her suffering and dying. On the other hand, even though transplantology does not fall under the CC in Poland the reason why some respondents declared that HCPs should have the right to invoke the CC in the context of transplantology is related to the ethical issues surrounding brain death and organ donation. The reason for this is that some individuals reject the definition of cerebral death and believe that it not necessarily permanent and irreversible, and therefore cannot determine the biological death of a person.

3 Tysiąc v. Poland concerned a visually impaired woman who was denied access to a legal abortion, even though medical diagnoses confirmed that continuing her pregnancy could severely impact her vision. Moreover, as her eyesight badly deteriorated after the delivery it was impossible to make corrective surgery and as a result, she faced a serious risk of developing blindness. Consequently, in March 2007 the Court ruled that the Polish government had failed to fulfil its obligation to ensure Tysiąc’s right in respect to private life and awarded her 39,000 euros for her pain, suffering, and legal fees. R.R. v. Poland concerned a woman who at 18 weeks of pregnancy was denied a referral to prenatal diagnostic examinations that would determine whether there was a severe foetal impairment. When she was finally informed of the test results, in accordance with Polish law, it was too late to perform a legal abortion as the foetus had already reached viability. Because R.R. had to continue her pregnancy to full term, she filed a complaint before the ECHR in 2004 and was awarded 65,000 euros of compensation. P. and S. v. Poland concerned 14-year-old P. who become pregnant as a result of rape but was denied a timely abortion even though it was lawful as the pregnancy resulted from a criminal act. Moreover, as P.’s mother S. was accused of forcing P. to have an abortion, family court placed P. in a juvenile shelter and S. was restricted in her parental rights. As a result of the court’s decision Poland was ordered to pay 61,000 euros.

4 It was initiated by Polish physician, Dr. Wanda Półtawska, who was a close collaborator of Pope John Paul II, whom she influenced on such topics as sexuality and contraception.
